# Genome Mining Approach Reveals the Occurrence and Diversity Pattern of Clustered Regularly Interspaced Short Palindromic Repeats/CRISPR-Associated Systems in *Lactobacillus brevis* Strains

**DOI:** 10.3389/fmicb.2022.911706

**Published:** 2022-06-03

**Authors:** Bahman Panahi, Mohammad Majidi, Mohammad Amin Hejazi

**Affiliations:** ^1^Department of Genomics, Branch for Northwest and West Region, Agricultural Biotechnology Research Institute of Iran (ABRII), Agricultural Research, Education and Extension Organization (AREEO), Tabriz, Iran; ^2^Department of Biotechnology, Faculty of Agriculture, Azarbaijan Shahid Madani University, Tabriz, Iran; ^3^Department of Food Biotechnoology, Branch for Northwest and West Region, Agricultural Biotechnology Research Institute of Iran (ABRII), Agricultural Research, Education and Extension Organization (AREEO), Tabriz, Iran

**Keywords:** CRISPR, diversity, evolution, genome, phage

## Abstract

Clustered regularly interspaced short palindromic repeats (CRISPR) together with their CRISPR-associated (Cas) genes are widely distributed in prokaryotes that provide an adaptive defense mechanism against foreign invasive DNA. There is relatively little knowledge about the CRISPR-Cas diversity and evolution in *Lactobacillus brevis* strains. Therefore, in this study, a genome-mining approach was employed to investigate the diversity and occurrence of the CRISPR-Cas system in 83 *L. brevis* strains. Moreover, trans-activating CRISPR RNA (tracrRNA) and protospacer adjacent motif (PAM) as pivotal elements for the successful targeting and inference of phages by the subtype II CRISPR-Cas systems were surveyed. Finally, evolutionary paths of *L. brevis* strains under selective pressure from foreign invasive DNA such as plasmids and phages of studied strains were surveyed using acquisition and deletion events analysis of spacers. A total of 127 confirmed CRISPRs were identified, which were distributed in 69 strains. Among strains with confirmed CRISPRs, 35 strains only contained one CRISPR locus, 23 strains contained two CRISPR loci, and 12 strains contained three to six CRISPR loci. *L. brevis* strains frequently harbor more than one CRISPR system. Analysis of confirmed CRISPR arrays showed that 31 out of 127 confirmed CRISPRs included Cas genes which were categorized as one of the II-A, II-C, and I-E subtypes. Analysis of subtype II-A spacers reflected divergent evolution for 18 strains into 16 unique groups. Additional analysis of spacer sequences also confirmed the implication of characterizing CRISPR-Cas systems in targeting of phages and plasmids. The current study highlighted the potential of utilizing CRISPR spacer polymorphism in genotyping lactobacillus strains. Moreover, it provides deep insights into the occurrence, diversity, and functional impacts of the CRISPR-Cas system in *L. brevis* strains.

## Introduction

Phages infect the bacterial hosts and highjack the host-cell machinery in the lytic (or virulent) lifestyle for replicating as well as destroying the host, which consequently led to decrease of living bacteria account, and even fermentation and production failure (Nami et al., 2021; Sadeghi et al., 2022). Other detrimental effects of LAB lysis by phages are the decline of acidification, texture development, and flavor enhancement. Because of thermal and pasteurization resistance of bacteriophages infecting lactic acid bacteria, completely removing phages from different contexts is impossible (Atamer et al., 2011; Nami et al., 2020). Consequently, there is a risk that phages multiply to high numbers, leading to considerable economic losses during the production process.

It has been revealed that clustered regularly interspaced short palindromic repeats (CRISPR) together with their CRISPR-associated (Cas) genes serve as an adaptive defense mechanism against phages by integration, transcription and degradation process (Jiang et al., 2016). Upon phage invading into the bacteria, a fragment of phage DNA is incorporated between conserved repeats of the CRISPR array (Marraffini and Sontheimer, 2010). Then, the entire CRISPR array is transcribed into a single RNA transcript and processed into an interfering unit with the help of Cas proteins, during the expression phase (Makarova et al., 2011). Ultimately, during the interference phase, the mature transcript, called CRISPR RNAs (crRNAs), is used to guide the Cas protein complex to cleave phage DNA in a sequence-specific manner upon reinfection (Van der Oost et al., 2009). Cas proteins are able to distinguish invaders through the occurrence of a protospacer adjacent motif (PAM) on the DNA of foreign targets (Crawley et al., 2018).

Clustered regularly interspaced short palindromic repeats/CRISPR-associated system has been divided into 2 classes, 6 types, and 33 subtypes (Koonin et al., 2017). In class 1, including type I, type III, and type IV, interference is carried out with large CRISPR associated complex for Cascade protein complex composed of multiple Cas proteins namely, Cas3 (sometimes fused to Cas2), Cas5–Cas8, Cas10, and Cas11, in different combinations, depending on the type and subtype (Koonin et al., 2017; Makarova et al., 2020; Khan et al., 2022). The mentioned Cas proteins form the interference or effector module, which is involved in target recognition and nucleic acid cleavage in CRISPR-Cas systems (Makarova et al., 2020). By contrast, in class 2 systems, including type II, type V, and type VI, the effector module is represented by a single, large protein Cas9, Cas12, or Cas13. According to previous reports, cas2 is also key gene for adaptation (Makarova et al., 2020).

Identifying the source of the spacer sequences, called the protospacer sequence, can tell a story of host-prey dynamics in different environment containing phage and invasive DNA elements (Levin et al., 2013). Iterative phage infection and consequent mutations to escape CRISPR targeting drive phage genome evolution as well as host strain evolution through adaptation and acquisition of new immunity-conferring spacers at CRISPR loci (Mahony and van Sinderen, 2015).

Numerous studies try to explore the occurrence and diversity of the CRISPR-Cas systems in different bacteria such as *Streptococcus thermophilus* (Horvath et al., 2008), *Sulfolobus islandicus* (Held et al., 2010), *Pseudomonas aeruginosa* (van Belkum et al., 2015), *Bifidobacterium longum* (Hidalgo-Cantabrana et al., 2017), *Staphylococci* (Rossi et al., 2017), *Lactobacillus curvatus*, *Lactobacillus graminis*, *Lactobacillus fuchuensis*, and *Lactobacillus sakei* (Ilıkkan, 2021) have been characterized. Recently, Yang et al. (2020) have investigated the occurrence and diversity of CRISPR loci in the *Lactobacillus casei* group. However, there is relatively little knowledge about the CRISPR-Cas diversity and evolution in *L. brevis* strains.

*Lactobacillus brevis* is the representative heterogeneous group of the LAB, which are catalase-negative, non-sporulating, non-motile, rod or coccus-shaped Gram-positive bacteria. It is a fastidious hetero-fermentative bacterium, which grows optimally at 30°C and within a pH range of 4–6 (Aasen et al., 2000; Murga et al., 2000). Among LAB, *L. brevis* have been granted Qualified Presumption of Safety (QPS) as a safety assessment tool for microorganisms added to food and feed (Fukao et al., 2009; EFSA FEEDAP Panel, 2014). Furthermore, it was found that this species exhibits probiotic characteristics in promoting gut microbiota fitness and consumer health (Bhavan, 2018). This is of particular interest for the utilization of *L. brevis* in the fermented food industry. Therefore, in this study, a genome-mining approach was employed to investigate the diversity and occurrence of the CRISPR-Cas system in *L. brevis* strains.

## Materials and Methods

### Data Collection and Clustered Regularly Interspaced Short Palindromic Repeats/CRISPR-Associated System Identification

The 83 genome sequences of *L. brevis* strains were retrieved and downloaded from National Center for Biotechnology Information (NCBI)^1^ Genbank. Subsequently, according to the feature and occurrence frequency of repeat sequences within one single CRISPR locus, CRISPRFinder web server^2^ categorizes CRISPR loci in two ‘‘Questionable’’ and ‘‘Confirmed’’ groups. Following steps were followed: (1) CRISPR possible localization, (2) identification of candidate direct repeat, (3) CRISPR structure validation, and (4) tandem repeat elimination and final introduction of CRISPR loci candidates. For parsing the unique CRISPR loci in each strain, data was subjected to CRISPRone server.^3^ Moreover, the CRISPRone were used for detection of false and mock CRISPRs, which may be tandem repeats, STAR-like elements, and others. Furthermore, to determine the presence and content of Cas genes and introduction of complete CRISPR-Cas systems, 10,000 bp upstream and 10,000 downstream of confirmed CRISPR loci were extracted from the corresponding genome and subjected to CRISPRCas Finder web server^4^ (Couvin et al., 2018). Furthermore, the BLAST (McGinnis and Madden, 2004) program was used to confirm type designation using signature of cas genes. Type designation manually was confirmed by the presence of cas3, cas9, and cas10 for Type I, II, and III, respectively.

### Repeat Structure Prediction

Secondary structures of RNA were predicted by the RNA fold Web server (Gruber et al., 2008), using minimum free energy (MFE) algorithm following default parameters. This server includes an implementation of the partition function for computing base pair probabilities and circular RNA folding (Hofacker, 2003).

Graphical representation of CRISPR loci and spacers/repeat array pattern visualization were performed using CRISPRVIZ tools (Nethery and Barrangou, 2019). This tool compares nucleotide spacer sequences present in a corresponding genome and then clusters them based on sequence similarity to assign a meaningful representative color for highlighting shared and unique spacer combinations.

### Protospacer Adjacent Motif and Target Analysis

Protospacers were identified using CRISPR target web server^5^ and BLASTn against plasmid and phage genomes. The protospacer with more than 85% identity and less than 3 mismatches were considered. To determine the PAM, 10 nucleotide flanking regions on the 5′ and 3′ ends of the protospacer sequences were aligned and representative logo were generated by WebLogo server (Crooks et al., 2004) for subtypes II and I, respectively.

### Trans-Activating CRISPR RNA Prediction

The trans-activating CRISPR RNA (tracrRNA) sequences were determined using PSI-BLAST with an *e*-value cut-off of 1e−06 using the multiple-alignments of Cas9-family proteins to identify anti-repeat region in the respective genomes per the strains with subtype II-A and II-C CRISPR-Cas systems as previously described by Briner et al. (2014).

### Phylogenetic Analyses

Multiple alignments of Cas1 amino acid sequences in *L. brevis* strain were performed using MSA (version 1.18.0) (Bodenhofer et al., 2015) R package. Analyses of phylogenetic and evolution were performed and compared with Cas1 proteins of CRISPR-Cas systems of *Lactobacillus bulgaricus* and *Lactobacillus johnsonii* using ape (version 5.0) (Paradis and Schliep, 2019) R package with neighbor joining algorithm.

## Results and Discussion

### Occurrence of the Clustered Regularly Interspaced Short Palindromic Repeats/CRISPR-Associated Systems in *Lactobacillus brevis* Strains

Identification and characterization of CRISPR-Cas systems in different bacteria have been noted by prior studies (Van Der Oost et al., 2014; Westra et al., 2014; Hidalgo-Cantabrana et al., 2019). Therefore, this study set out with the aim of the characterization of CRISPR-Cas systems in *L. brevis* strains. As mentioned in the method and material section, available genomes of 85 *L. brevis* strains, including 26 complete and 59 draft genomes were analyzed for occurrence and diversity pattern exploring of CRISPR systems (Supplementary Table 1). A total of 127 and 280 confirmed and questionable CRISPRs were identified in analyzed genomes, respectively. The confirmed CRISPRs were distributed in 69 strains which accounted for 81% of *L. brevis* strains. CRISPR-Cas occurrence rate of *L. brevis* strains estimated about 27%, which were lower than the estimated occurrence rate for *L. casei* (39%) and other bacteria (45%) (Grissa et al., 2007; Yang et al., 2020). Among strains with confirmed CRISPRs, 35 strains only contained one CRISPR locus, 23 strains contained two CRISPR loci, and 12 strains contained three to six CRISPR loci. This may be related to hypervariability in genomes of *L. brevis* strains which enables notably adaptation to the different milieus (Feyereisen et al., 2019).

More details of confirmed CRISPR loci in each corresponding strain are provided in Supplementary Table 2. CRISPR-Cas system was confirmed by a series of Cas genes flanked by CRISPR arrays. CRISPR-Cas Finder defines these complete CRISPR-Cas systems to subtypes based on the Cas genes. However, some confirmed CRISPR arrays uncoupled with Cas genes were defined as orphan CRISPR. Analysis of confirmed CRISPR arrays using CRISPR-Cas Finder showed that 31 out of 127 confirmed CRISPRs included Cas genes which were categorized as one of the II-A, II-C, and I-E subtypes based on the Cas genes signature (Table 1). It is in agreement with the previous finding which was reported that lactic acid bacteria spanned three distinct subtypes of CRISPR-Cas systems (Horvath et al., 2008). Complete CRISPR-Cas system distributed in 23 strains. Most of the strains only had one subtype, based on the Cas genes signature, however, eight strains were found that harbor two subtypes of CRISPR-Cas system according to the Cas genes signature, of which 3M004, BIO5542, A, HF01, and Q strains had subtypes II-A and II-C, ZLB004 and TR055 strains had subtypes II-A and I-E, and TUCO-5E had subtypes II-C and I-E (Table 1).

**TABLE 1:** Clustered regularly interspaced short palindromic repeats/CRISPR-associated system in *L. brevis* strains with confirmed CRISPR array.

Strains	CRISPR/Cas type	Direction	Strains	CRISPR/Cas type	Direction
ATCC 367	Orphan	–	UCCLBBS449	Orphan	–
KB290	Orphan	–	UCCLB521	Orphan	–
EW 136.LBL	Orphan	–	TR169	Orphan	–
AG48	Orphan	–	TR052	TypeII-A	Negative
WK12	Orphan	–	TR055	TypeII-A	Negative
BSO	Orphan	–	TR055	TypeI-E	Negative
DmCS_003	Orphan	–	NBRC 3960	Orphan	–
TMW 1.465	Orphan	–	NBRC 12005	Orphan	–
TMW 1.313	Orphan	–	NBRC 13110	Orphan	–
47f	Orphan	–	LB_AVK	TypeII-A	Negative
Lb1595	Orphan	–	BIO5542	TypeII-A	Positive
D6	Orphan	–	BIO5542	TypeII-C	Positive
NPS-QW-145	TypeII-A	Positive	HQ1-1	Orphan	–
DPC 6108	Orphan	–	Dm-2019-67	Orphan	–
BSO 310	Orphan	–	Dm-2019-70	Orphan	–
EF	Orphan	–	Dm-2019-31	Orphan	–
G101	TypeI-E	Positive	LBH1073	TypeII-A	Negative
CRL 2013	Orphan	–	MCC633	TypeI-E	Positive
TMW 1.2112	TypeII-A	Positive	N38	Orphan	–
TMW 1.2113	TypeII-A	Positive	G430	Orphan	–
TMW 1.2108	Orphan	–	Isolate A	TypeII-A	Positive
TMW 1.2111	Orphan	–	Isolate A	TypeII-C	Positive
100D8	Orphan	–	HF01	TypeII-A	Positive
SRCM101174	Orphan	–	HF01	TypeII-C	Positive
MGYG-HGUT	Orphan	–	JK09	Orphan	–
SRCM101106	TypeII-A	Positive	Isolate Q	TypeII-A	Positive
3M004	TypeII-A	Positive	Isolate Q	TypeII-C	Positive
3M004	TypeII-C	Positive	TUCO-5E	TypeII-C	Negative
SF15B	Orphan	–	TUCO-5E	TypeI-E	Negative
ZG1	TypeII-A	Positive	WIKIM12	Orphan	–
SF9B	Orphan	–	KMB_615	TypeI-E	Positive
BDGP6	TypeII-A	Positive	KMB_620	Orphan	–
NBRC 3345	TypeII-A	Negative	CD0817	TypeII-A	Positive
DS1_5	Orphan	–	LMT1-73	Orphan	–
DM9218	Orphan	–	SRCM 103306	Orphan	–
ZLB004	TypeII-A	Negative	UCCLBBS124	Orphan	–
ZLB004	TypeI-E	Positive	SA-C12	Orphan	–
NCTC13386	TypeII-A	Positive	UCCLB556	Orphan	–
UCCLB95	Orphan	–			

### Diversity of the Clustered Regularly Interspaced Short Palindromic Repeats/CRISPR-Associated System in *Lactobacillus brevis* Strains

**FIGURE 1:**
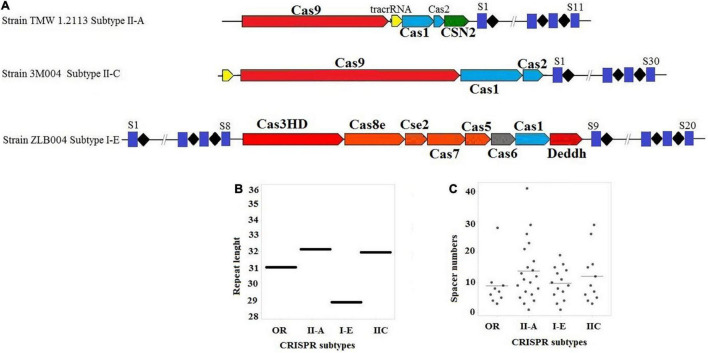
**(A)** Schematic diagram of complete CRISPR-Cas array; **(B)** distribution of the repeat length, in nucleotides for each CRISPR-Cas subtype; **(C)** distribution of spacer numbers for each CRISPR-Cas subtype. Trans-activating CRISPR (tracr) RNA for subtype II systems is shown in yellow.

Among the strains with a complete CRISPR-Cas system, nineteen *L. brevis* strains contained subtype II-A, six strains contained subtype II-C, and six strains contained subtype I-E. As shown in Figures 1A, subtype II-A encoded *cas9*, *cas1*, *cas2*, and signature cas gene (*csn2*). In comparison with subtype II-A, the subtype II-C encoded *cas9*, *cas1*, and *cas2* genes with different patterns. Regarding the subtype I-E, *cas3HD*, *cas8e*, *cse2*, *cas7*, *cas5*, *cas6*, *cas1*, and *Deddh* were encoded by the *L. brevis* strains (Figure 1A).

Figure 1B presents the average length of the repeat sequences for subtype II-A, I-E, and II-C which were 32, 28, and 32 nucleotides, respectively. The lengths of repeat sequences of subtype II-A found for *L. brevis* was slightly lower than *L. casei* (Yang et al., 2020).

It can be seen from the data in Figure 1C that the size of the repeat-spacer arrays found in *L. brevis* is diverse, and ranging from one to 41 spacers (Supplementary Table 3). The average numbers of spacers in the orphan CRISPR systems were nine spacers per locus. In subtype I-E, the average numbers of spacers in the CRISPR system were ten spacers per locus with some loci encoding 20 unique genetic immunization events. Subtype II-C were 11 spacers per locus with a range of 3–29 spacers. The number of CRISPR spacers in subtype II-A systems is, on average, greater than subtype II-C, I-E, and orphan systems, averaging 12 spacers per locus, but can reach as many as 41 spacers (Figure 1C and Supplementary Table 3). However, the findings of the current study do not support the previous research in *L. casei* groups and Bifidobacterium, which showed that the variability of spacers in subtype I-E CRISPR-Cas system was higher than other subtypes (Briner et al., 2015; Yang et al., 2020). Turning now to the evidence on size and rate of occurrence of subtype II-A, we thought that this subtype is the most active loci in *L. brevis* genome. The present findings seem to be inconsistent with other research which hypothesized that subtype I-E CRISPR-Cas in Bifidobacterium and *L. casei* (Briner et al., 2015; Yang et al., 2020) and I-B/Tneap subtype in *Clostridium perfringens* (Long et al., 2019) are the most active systems against foreign DNA and phages.

**FIGURE 2:**
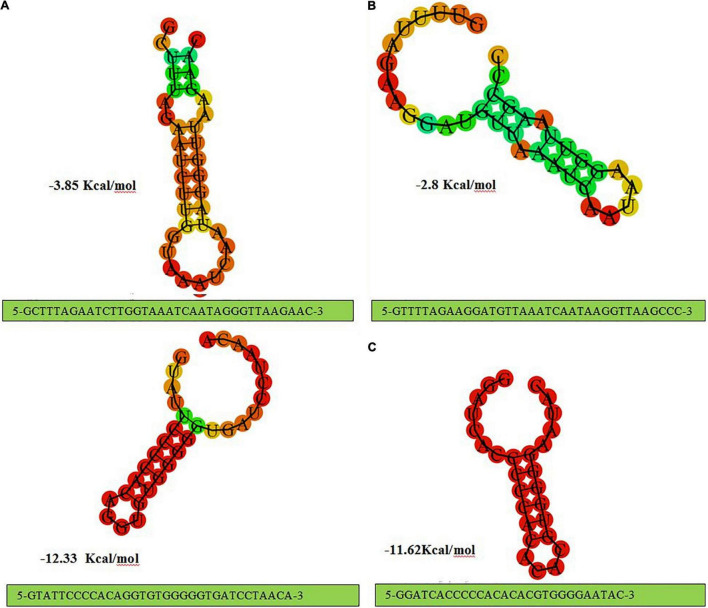
The prediction of consensus direct repeat secondary structure and corresponding MFE values in type II-A **(A)**; type II-C **(B)**; and type I-E **(C)**.

Structural analysis of direct repeat also provides insight regarding CRISPR -Cas systems in *L. brevis* strains. The present findings seem to be consistent with a previous study in the *L. casei* group, which found that the stems were created in the middle of RNA secondary structures (Yang et al., 2020). According to the predicted structures depicted in Figure 2, CRISPR loci which associated with Cas genes subtype II-A direct repeat sequence included 7 bp stem length (Figure 2A), 8 bp for subtype II-C direct repeat sequence (Figure 2B), 7 and 6 bp for subtype I-E direct repeat sequence (Figure 2C). Considering the conservation and stability, predicted secondary structures of CRISPR loci associated with subtype II-A and II-C Cas signatures had the shortest stem with the lowest MFE values. Compared with the subtype II CRISPR system, subtype I-E had larger MFE with approximately similar stem lengths. It has been demonstrated that the structures with higher GC content and longer stems are more stable than structures with lower GC content and shorter stems (Yang et al., 2020). According to the default parameters adjusted by the system, red and green regions indicated the high and low probability of conformation in each structure.

**FIGURE 3:**
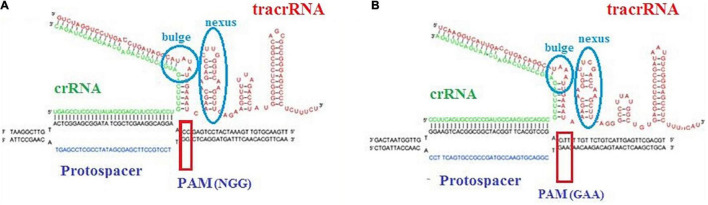
Representative crRNA:tracrRNA duplex binds with target DNA sequence next to PAM sequence in CRISPR-Cas subtype II-A **(A)** and subtype II-C **(B)**.

Trans-activating CRISPR RNA prediction in the *L. brevis* strains that carry the subtype II system led to identification of anti-repeat sequences in each of the dissected CRISPR systems (Figure 1A). Details of identified anti-repeat sequences including position in genome, strand, match, and mismatch number are presented in Supplementary Table 4. Moreover, crRNA:tracrRNA duplexes were presented in Figure 3.

Our analysis showed that the predicted tracrRNA is located between cas9 and cas1 on the minus strand in NPS-QW-145, ZG1, NBRC 3345, ZLB004, TR052, TR055, LB_AVK, LBH1073, and TUCO-5E strains and on the plus strand in TMW 1.2112, TMW 1.2113, SRCM101106, 3M004, BDGP6, NCTC13386, and BIO5542 strains. Moreover, the tracrRNA in the II-C system, found in 3M004, is upstream of the 5′ end of cas9 on the positive strand. Our findings were in line with the previous finding in Bifidobacterium (Briner et al., 2015).

**FIGURE 4:**
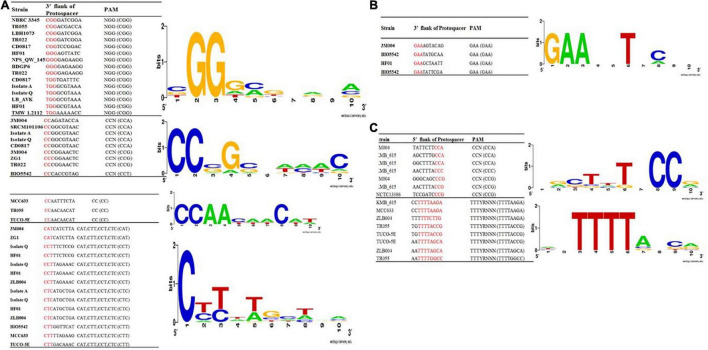
Predicted PAMs motifs in *L. brevis* strain for strains which harbored the subtype II **(A)**, II-C **(B)**, and I-E **(C)**. The height of each letter represents the conservation of that nucleotide at each position. PAM motifs were extracted from the 10 nt flank at the 3′ end for subtypes II-A **(A)** and subtypes II-C **(B)** and from the 10 nt flank on the 5′ end for subtype I-E **(C)** of the predicted protospacers. Red color highlighted nucleotides in the given sequences are the consensus sequence of predicted PAM motifs in each strains.

Protospacer adjacent motif is another functionally critical element in CRISPR-Cas systems. It has been demonstrated that this motif used by the Cas proteins to bind phages and other foreign invasive then interrogates the DNA from 3′ to 5′ DNA (Mojica et al., 2009). Identified PAM motifs, based on all identified phages and plasmids which provided in Supplementary Figures 1, 2, for subtypes II and I and corresponding strains were presented in Figure 4A. As presented in Figure 4A, for subtype II-A, four types of PAM motifs including 5′-NGG-3′, 5′-CCN-3′, 5′-CC-3′, 5′-CAT, CTT, CCT, and CTC-3′ were identified for *L. brevis* flanking the 3′ end of protospacers. 5′-NGG-3′ motifs that were the most dominant motif in subtype II array, identified in NBRC 3345, TR055, LBH1073, TR022, CD0817, HF01, NPS_QW_145, BDGP6, Isolate A, Isolate Q, and HF01 strains. The identified PAM, 5′-NGG-3′, is the same as the well-characterized *Streptococcus pyogenes* and Bifidobacterium PAM (Figure 4A). Regarding the subtype II-C, we found only one type of PAM motif, 5′-GAA-3′, flanking the 3′ end of protospacers (Figure 4B). In comparison, 5′-CC-3′ motif found only in three strains including MCC633, TR055, and TUCO-5E. Regarding the PAM motif in 5′ flank of protospacers for subtype I-E, two motifs including 5′-TTTYRNNN-3′ and 5′-CCN-3′ were detected (Figure 4C).

**FIGURE 5:**
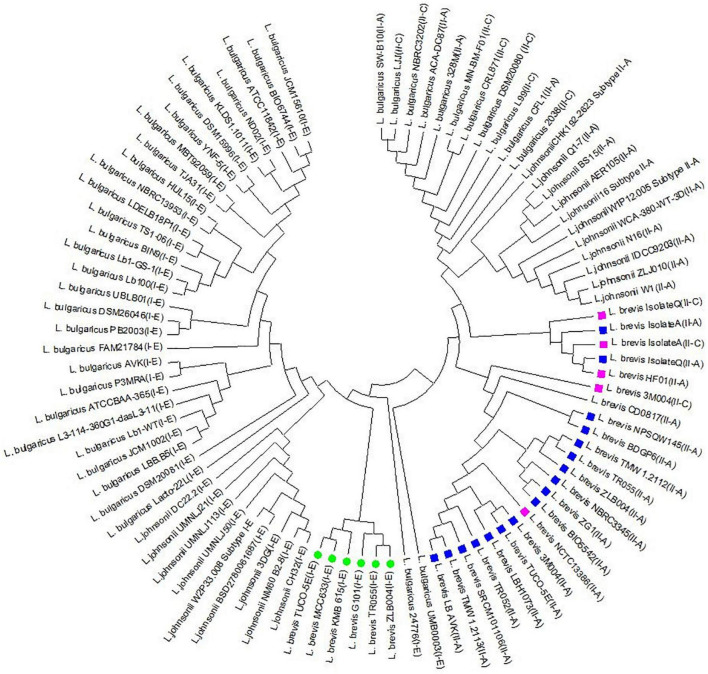
Phylogeny tree based on Cas1 amino acid sequence in subtype II-A, II-C, and I-E related to *L. brevis*, *L. johnsonii*, and *L. bulgaricus.* Green circle, purple box, and blue box indicate the I-E, II-C, and II-A subtypes in *L. brevis* strains, respectively.

To gain insights into the level of conservation and divergence within each subtype of CRISPR-Cas systems, multiple alignments and phylogeny analysis were performed based on Cas1 sequences. The Cas1 tree revealed three major clusters that correspond to different subtypes and three sub clusters for subtype II-A. Nevertheless, the strains with similar spacer content such as ZG1, NBRC3345 were clustered in the same clusters that were created based on the Cas1 sequence. Similar findings were also observed related to TMW 1.2112 and TMW 1.2113 (Figure 5).

The current study indicates that there is great diversity between the three CRISPR-Cas systems identified in *L. brevis* strains genomes at CRISPR repeat sequence; the Cas gene sequence and organization; spacer content and pattern and CRISPR-Cas architecture. Such diverse CRISPR-Cas systems indicate that these systems are naturally active and important in *L. brevis* in terms of adaptive immunity and evolutionary relationship. These findings are consistent with those of Crawley et al. (2018) and Yang et al. (2020) who showed that CRISPR-Cas systems components vary at the strain level in most Lactobacillus species. The diversity of Cas1 sequences has occasionally been seen naturally in *Staphylococcus thermophilus* (Wei et al., 2015) and *Haloarcula hispanica* (Li et al., 2014).

### Genotyping Through Clustered Regularly Interspaced Short Palindromic Repeats Spacer Analysis

**FIGURE 6:**
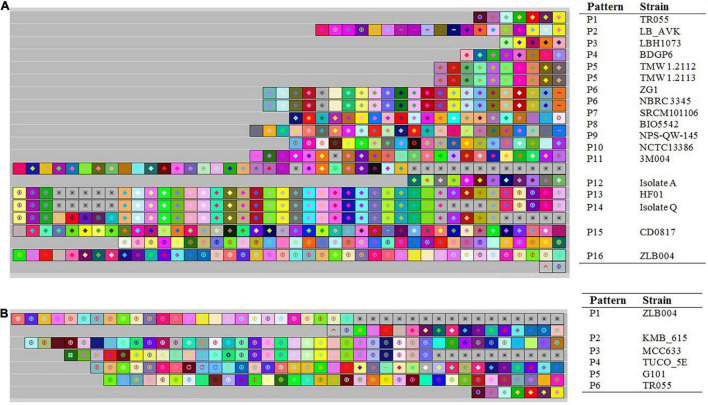
Graphical representation of spacer pattern in subtypes II-A **(A)** and I-E **(B)** CRISPR-Cas systems. Gray squares containing an “X” represent no spacers. Each color combination represented a unique spacer sequence based on the nucleotide sequences. The newly and earliest acquired spacers are shown on the left and right sides, respectively.

Evolutionary paths of *L. brevis* strains under selective pressure from foreign invasive DNA such as plasmids and phages of studied strains were studied using acquisition and deletion events analysis of spacers. In this survey, the spacers were arranged from the ancestral end toward the recently acquired end (left), and each color combination represented a unique spacer sequence based on the nucleotide sequences (Yang et al., 2020). Ancestral spacers are on the right-hand side of the figure (Figure 6). Moreover, each color combination represented a unique spacer sequence based on the nucleotide sequences. From the data in Figure 6, it is apparent that NBRC 3345 with ZG1 and TMW 1.2112 with TMW 1.2113 strains shared a completely similar pattern of composition, acquisition, and deletion events of spacers in the evolutionary period, while other strains had different later acquired spacers. Strains with a similar pattern of spacers may be originated from a shared ancestral, therefore we deduced that the NBRC 3345 and ZG1 originated form same ancestral. Moreover, TMW 1.2112 with TMW 1.2113, TR055 with LB_AVK, and HF01 with isolateQ are showing common lineages.

Analysis of spacer composition and acquisition events in the spacer of subtype I-E CRISPR-Cas system is shown in Figure 6B. According to the spacer alignment, the strain with subtype I-E had a unique pattern of the spacer in their CRISPR-Cas systems indicating that the strains had different later acquired spacers. All strains in subtype I-E are showing common lineages originated from different ancestors.

It has been demonstrated that the strain with a similar pattern of spacers may be initially exposed to the same environment and originated from a shared ancestor (Long et al., 2019). In this regards, we dissected the isolated sources of the strains with each CRISPR-Cas subtypes (Supplementary Table 5). It is apparent that there is not significant correlation between CRISPR-Cas systems subtype and habitats in which strains were isolated. Nevertheless, the NBRC 3345 and ZG1 as well as TMW 1.2112 and TMW 1.2113, which isolated from similar milieus, harbored the same spacer’s pattern. This finding may be indicating that the strains with similar pattern of spacers initially exposed to the same environment. However, regarding the other above-mentioned strains which showing common lineages they were diverged after originating from same ancestral.

### Homology Analysis of Spacer Sequences

**FIGURE 7:**
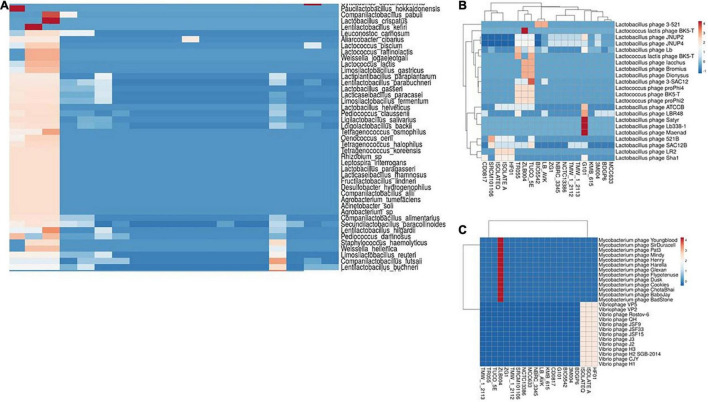
Cluster analysis of homology of spacer in *L. brevis* strains targeting with different classes of bacterial plasmids **(A)**, Lactobacillus phages **(B)**, and Mycobacterium and Vibrio phages **(C)**. Each box showing the presence of target phages and the color range indicates the frequency of corresponding targets. Blue to red indicates the low number to high number.

Homology analysis of spacer sequences of CRISPR systems with available phage and plasmid sequences could be helpful for elucidation of the immunity systems complexity of studied strains. Among the 23 strains of *L. brevis*, which had complete CRISPR-Cas systems, 19 strains showed at least one spacer targeting plasmids (Supplementary Figure 1). LBH1073, TR052, and BDGP9 strains not show any spacer targeting plasmids. Among the strains with spacer targeting plasmids, ZLB004 strain, showed higher diversity of targeting plasmids (99 types of plasmids), whereas TMW_1_2113 and TMW_1_2112 showed only one spacer targeting plasmid (plasmid of *Arthrobacter crystallopoietes*) in their spacer sequences. The most frequently matched events happened with the plasmid sequences from *Lactiplantibacillus plantarum*, which was consistent with target of total plasmid. This result is consistent with prior findings in the *L. casei* group (Yang et al., 2020). Results of our study also showed that the most frequent match events happened with the plasmid sequences from Lactobacillus and Staphylococcus (Figure 7A). Moreover, our finding demonstrated that the strains with type-II CRISPR systems presented more spacer targeting foreign plasmids. However, the findings of the current study do not support the previous research in the *L. casei* group (Yang et al., 2020).

Homology analysis of spacer sequences with phage genomes also revealed that the spacer of 20 strains had at least one spacer targeting phages. Results of homology analysis revealed that *L. brevis* strains majority targeting by four groups of phages including Lactobacillus phages, Streptococcus phages, Vibrio phages, and Mycobacterium phages (Supplementary Figure 2). It is apparent from Figure 6B, Lactobacillus phage Satyr, Lactobacillus phage Lb 338-1 and Lactobacillus phage Maenad specifically targeted by the strain G101 spacers. Moreover, *Lactococcus lactis* phage BK5-T and groups of Mycobacterium phages are specifically targeted by spacers of ZLB004 strain (Figure 7B). On the other hand, vibrio phages are only targeted by the spacers of HF01, IsolateA, and IsolateQ strains (Figure 7C). Data presented in Supplementary Figure 2 also revealed the greatest number of phages targeted by spacers of *L. brevis* G101 and *L. brevis* ZLB004, in comparison with other strains. In *L. brevis* G101, the spacer sequences match to 75 streptococcus phages. A similar pattern of frequency of commonly targeted phages between strains related to different subtypes of CRISPR-Cas systems was observed. As depicted in Supplementary Figure 2, in all of the strains related to three CRISPR subtypes, Lactobacillus phage JNUP4 and Lactobacillus phage JNUP2 followed by Lactobacillus phage Lb and Lactobacillus phage SAC12B were the most commonly targeting phages. Nevertheless, the strain specific targeted phages in subtype I-E are more frequent than subtype II-A and II-C. Spacer sequences that showed positive matches to various phages in genomic data suggest that *L. brevis* requires defense systems to fight against phages (Briner et al., 2015). In line with this hypothesis, dissection in *L. brevis* strain TR055 showed that some spacers shared a region with the gene encoding tail component. These components are key elements in phage replication, indicating that one of the immunity strategies of *L. brevis* strains by the CRISPR-Cas system potentially interfere with the replication process.

## Conclusion

In conclusion, the findings of the current study revealed that *L. brevis* strains had a relatively great diversity of CRISPR-Cas systems, including three subtypes, with varying CRISPR-Cas components and architecture, highlighting that these systems may be naturally active immune systems against phage and foreign invasive DNA. Spacer homology analysis also confirmed the implication of characterized CRISPR-Cas systems in targeting of phages and plasmids. In addition, our study highlighted the potentiality of CRISPR spacer polymorphism for harnessing in genotyping of lactobacillus strains and designing of new genome editing platforms. This study also provides deep insights into the occurrence, diversity, and functional impacts of the CRISPR-Cas system in *L. brevis* strains.

## Data Availability Statement

The datasets presented in this study can be found in online repositories. The names of the repository/repositories and accession number(s) can be found in the article/Supplementary Material.

## Author Contributions

BP: designing experiment and data analysis. BP, MM, and MH: writing. All authors contributed to the article and approved the submitted version.

## Conflict of Interest

The authors declare that the research was conducted in the absence of any commercial or financial relationships that could be construed as a potential conflict of interest.

## Publisher’s Note

All claims expressed in this article are solely those of the authors and do not necessarily represent those of their affiliated organizations, or those of the publisher, the editors and the reviewers. Any product that may be evaluated in this article, or claim that may be made by its manufacturer, is not guaranteed or endorsed by the publisher.

## Footnotes

1
https://www.ncbi.nlm.nih.gov/

2
https://crispr.i2bc.paris-saclay.fr/Server/

3
https://omics.informatics.indiana.edu/CRISPRone/

4
https://crisprcas.i2bc.paris-saclay.fr/CrisprCasFinder/Index

5
http://crispr.otago.ac.nz/CRISPRTarget/crispr_analysis.html

## Supplementary Material

Supplementary Figure 1 Cluster analysis of homology of spacer in *L. brevis* strains targeting with different classes of bacterial plasmids.

Supplementary Figure 2 Cluster analysis of homology of spacer in *L. brevis* strains targeting with different classes of phages.
