# Supplementary figures and images for: Genome Mining Approach Reveals the Occurrence and Diversity Pattern of Clustered Regularly Interspaced Short Palindromic Repeats/CRISPR-Associated Systems in Lactobacillus brevis Strains
Source: Front Microbiol. 2022 Jun 3;13:911706. doi: 10.3389/fmicb.2022.911706 (PMC9204096; doi:10.3389/fmicb.2022.911706)

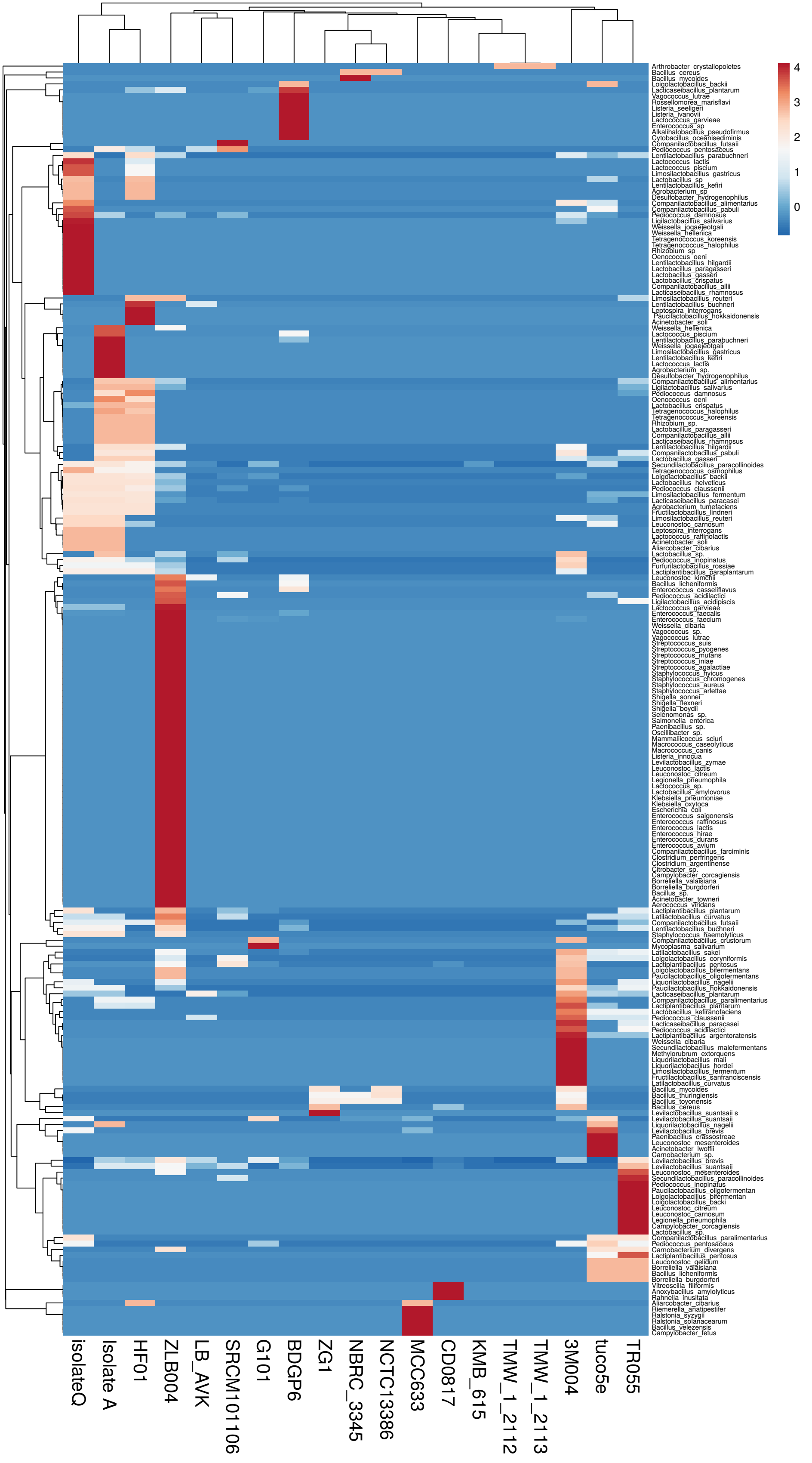

Supplement: Supplementary Figure 1 — Cluster analysis of homology of spacer in L. brevis strains targeting with different classes of bacterial plasmids. [file Data_Sheet_1.PDF]

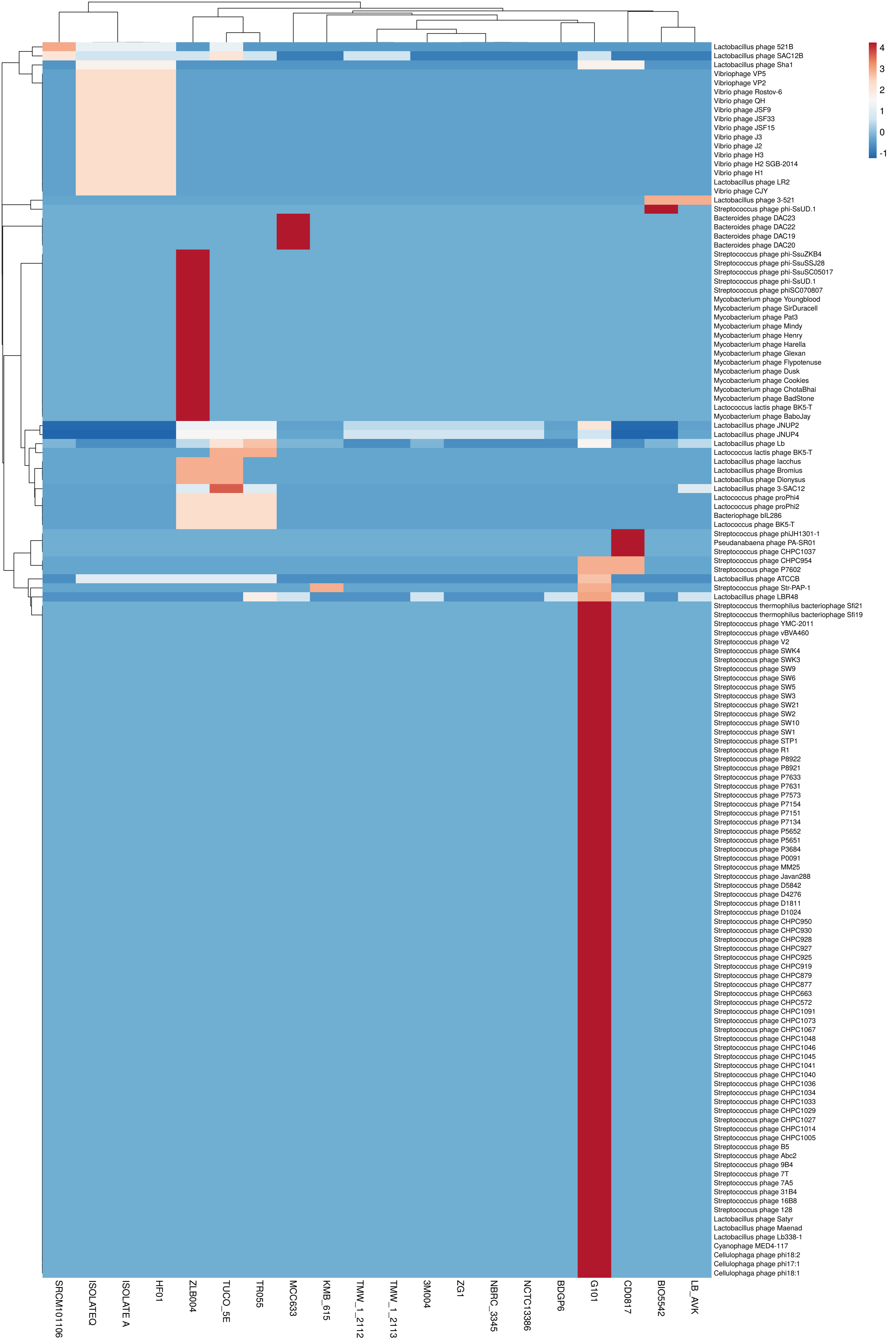

Supplement: Supplementary Figure 2 — Cluster analysis of homology of spacer in L. brevis strains targeting with different classes of phages. [file Data_Sheet_2.PDF]
